# Don't Get Too Comfortable: Destabilizing the Ground State to Speed a Reaction

**DOI:** 10.1371/journal.pbio.1001600

**Published:** 2013-07-02

**Authors:** Richard Robinson

**Affiliations:** Freelance Science Writer, Sherborn, Massachusetts, United States of America

[Fig pbio-1001600-g001]Imagine you are walking across a field, when you see a fence in the distance, blocking your way. OK, you say, I can probably hop over it. But as you come nearer, you find that on the near side of the fence, and running parallel to it, is a deep ditch. From the bottom of the ditch, you realize, the top of the fence will be just too high to jump. What to do?

**Figure pbio-1001600-g001:**
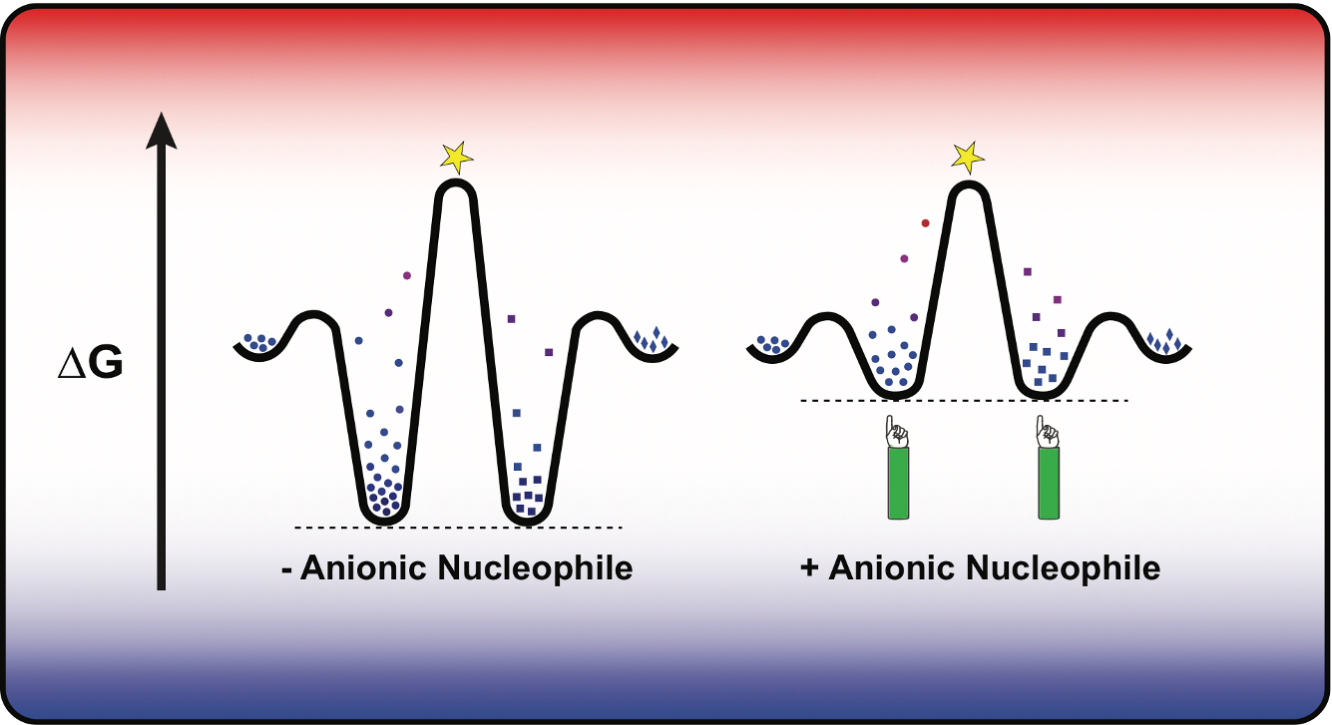
Electrostatic repulsion between a negatively charged substrate and an enzyme's anionic nucleophile, such as a negatively charged serine, can destabilize the ground state, reducing the energy needed to cross the reaction barrier.

For enzymatic reactions carried out by phosphatase enzymes, the answer is, fill the ditch. That's the conclusion from new work by Logan Andrews, Tim Fenn, and Daniel Herschlag, who show that a crucial element of these enzymes' catalytic mechanism is to destabilize the initial binding state of enzyme and substrate, in effect filling the energy ditch into which they would otherwise fall as they come together, and bringing them closer to the energy level needed to “hop the fence.” Their work further identifies the central atoms that contribute to this effect, and provides insight into how some phosphatases can at once be so prodigious in their enzymatic activity and so wide-ranging in their choice of targets.

Biochemists characterize the energetic changes during a chemical reaction with a simple and powerful graph, whose vertical axis indicates the overall energy of the molecules involved. When reactant molecules collide, some of their kinetic energy is absorbed as increased potential energy. This distorts their shapes, weakening their bonds and “pushing” them toward the transition state, the top of the energy fence at which the arrangement of atoms is neither reactant nor product but something in between. From there, they can complete the rearrangement, releasing energy as new bonds form, rolling downhill to become products. The graph of such a reaction has a single peak.

With an enzyme, though, the graph becomes more complex. Most importantly, an enzyme stabilizes the transition state, lowering the height of the fence. But the enzyme also binds to the reactant molecule (now called a substrate), lowering its energy as well. The graph has a trough, indicating the enzyme-substrate complex, followed by a peak (the enzyme-transition state complex), then another trough (the enzyme-product complex). If the binding between enzyme and substrate is too tight—if the ditch is too deep—the substrate will be unwilling to rearrange its atoms into the transition state.

Phosphatases cleave phosphate groups from biological compounds. Some phosphatases, including alkaline phosphatase, aren't picky about their substrates, because they don't bind to remote portions of the substrate molecule. Instead, enzyme and substrate engage each other exclusively through five oxygen atoms: four on the phosphate, and one on the enzyme. The incoming phosphate's four negatively charged oxygens find a lot to be attracted to at the active site, including two positively charged metal ions and several weakly positive hydrogens, all positioned to hold the phosphate neatly in place. And this is when they also encounter that fifth oxygen; it is part of a serine amino acid (serine102), and in its normal state it is itself negatively charged. Could this oxygen provide a destabilizing effect that promotes the formation of the transition state?

To test that idea, the authors mutated serine102 to either glycine or alanine, neither of which carry an oxygen or negative charge. They found that phosphate binds to the mutant enzyme so strongly that it takes longer than several days to leave. By mutating a second site to reduce phosphate's affinity, as well as manipulating pH and studying the various anionic forms of the phosphate ion in combination with the mutant and wild-type enzymes, they inferred that serine destabilized enzyme-substrate binding by a factor of more than 1,000-fold. The same serine also destabilized binding of enzyme to product (a ditch on the other side of the fence), preventing end-product inhibition of further enzyme activity.

Finally, they showed that a similar mechanism was likely at work in another class of enzyme, a protein tyrosine phosphatase, in which the critical amino acid is a negatively charged cysteine. Similar experimental manipulations suggested a destabilizing effect of the cysteine approximately equal in magnitude to that of the serine in alkaline phosphatase.

These results provide an important clue to one aspect of the mechanism of an essential group of enzymes. However, the authors note, phosphatases speed reactions by an astonishing 10^27^-fold versus the uncatalyzed reaction in water, meaning multiple other mechanisms are likely at work, including subtle positioning effects of the substrate by the geometry of the active site.

**Andrews LD, Fenn TD, Herschlag D (2013) Ground State Destabilization from Anionic Nucleophiles in Phosphoryl Transfer Active Sites and Femtomolar Ligand Affinity Revealed by Binding Studies. doi:10.1371/journal.pbio.1001599**

